# PD200 Implications Of The European Artificial Intelligence Act For Health Technology Assessment

**DOI:** 10.1017/S0266462324004227

**Published:** 2025-01-07

**Authors:** Rossella Di Bidino, Giuseppe Arbia, Dario Sacchini, Americo Cicchetti

## Abstract

**Introduction:**

The European parliament is working on a proposal for an Artificial Intelligence (AI) Act. The goal is to guarantee that AI systems used in the European Union are “safe, transparent, traceable, non-discriminatory and environmentally friendly.” The Act is not directly linked to health technology assessment (HTA), but implications for HTA are expected.

**Methods:**

The final aim of the AI Act is the adoption of harmonized rules for AI foundation models. Foundation models are designed to produce a wide variety of outputs that are being adopted across various sectors. Therefore, their adaptation to specific (clinical) needs plays a key role and has implications on the required training data. The goal of our analysis was to critically investigate the potential implications of the Act’s requirements for HTA. The analysis moves from the technology (foundation models in health care) to the requirements and adopted terminology.

**Results:**

The definition of methods to assess the adaptation of foundation models has become a priority for HTA. The Act focuses on data quality, transparency, human oversight, and accountability. Available frameworks, such as the one developed by the AI MIND project, include them, but experience is required. HTA must define how to adapt its methods and frameworks as well as the risk levels addressed by the Act. In addition, the reference to energy efficiency standards confirms the need for HTA to clarify the role of and methods for environmental impact assessment. Finally, the patient perspective has great relevance in relation to the risk of discrimination.

**Conclusions:**

The AI Act confirms the importance of topics already debated in HTA that still need resolution and testing. Harmonization of rules for AI and approaches for HTA is the main challenge. The AI Act, the HTA regulation, and ongoing European Union projects (i.e., AI-MIND and European Digital Health Technology Assessment) are showing the route to follow in the coming years.

